# 6-Gingerol attenuates hepatic ischemia/reperfusion injury through regulating MKP5-mediated P38/JNK pathway

**DOI:** 10.1038/s41598-024-58392-1

**Published:** 2024-04-02

**Authors:** Qiwen Yu, Jiye Li, Mengwei Cui, Chaopeng Mei, Qianqian He, Xiaoxiao Du

**Affiliations:** 1 Department of Emergency Medicine, The First Affiliated Hospital of Zhengzhou University, Zhengzhou, 450052 China; 2 Department of Hepatobiliary and Pancreatic Surgery, The First Affiliated Hospital of Zhengzhou University, 1 Jianshe East Road, Erqi, Zhengzhou, 450052 Henan China

**Keywords:** Hepatic ischemia/reperfusion injury, 6-Gingerol, MKP5, P38/JNK pathway, Liver, Liver diseases

## Abstract

6-Gingerol, the main bioactive compound of ginger, has antioxidant, anti-inflammatory, anti-cancer and neuroprotective effects. However, it is unclear whether 6-Gingerol has protective effects against hepatic ischemia/reperfusion (I/R) injury. In this study, the mouse liver I/R injury model and the mouse AML12 cell hypoxia/reoxygenation (H/R) model were established by pretreatment with 6-Gingerol at different concentrations to explore the potential effects of 6-Gingerol. Serum transaminase levels, liver necrotic area, cell viability, inflammatory response, and cell apoptosis were used to assess the effect of 6-Gingerol on hepatic I/R or cell H/R injury. Quantitative polymerase chain reaction (qPCR) and Western blotting were used to detect the mRNA and protein expression. The results show that 6-Gingerol decreased serum alanine aminotransferase (ALT), aspartate aminotransferase (AST) levels, liver necrosis, inflammatory cytokines *IL-1β, IL-6, MCP-1, TNF-α* expression, Ly6g+ inflammatory cell infiltration, protein phosphorylation of NF-κB signaling pathway, Terminal deoxynucleotidyl transferase dUTP nick-end labeling (TUNEL) positive cells, cell apoptosis rate, the protein expression of pro-apoptotic protein BAX and C-Caspase3, increased cell viability, and expression of anti-apoptotic protein BCL-2. Moreover, 6-Gingerol could increase the mRNA and protein expression of mitogen activated protein kinase phosphatase 5 (MKP5) and inhibit the activation of P38/JNK signaling pathway. In MKP5 knockout (KO) mice, the protective effect of 6-gingerol and the inhibition of P38/JNK pathway were significantly weakened. Therefore, our results suggest that 6-Gingerol exerts anti-inflammatory and anti-apoptotic effects to attenuate hepatic I/R injury by regulating the MKP5-mediated P38/JNK signaling pathway.

## Introduction

Hepatic I/R injury refers to the pathological process of ischemic injury in the liver caused by various factors that are aggravated by the restoration of blood supply^1^. Hepatic I/R injury is a critical cause of postoperative liver function decline, failure, or non-functioning of transplanted liver. In liver transplantation, hepatic I/R injury leads to 10% early organ failure and a high rate of acute and chronic rejection^2^. Thus, finding new therapeutic methods to reduce hepatic I/R injury is of great theoretical significance and clinical value.

In recent years, various approaches have been explored to mitigate hepatic I/R injury, such as ischemic preconditioning, gene targeting techniques and pharmaceutical interventions, with a growing emphasis on pharmacological interventions^3–5^. 6-Gingerol is the main bioactive compound of ginger, which has been extensively studied and shown to possess antioxidant, anti-inflammatory, anti-tumor, immunomodulatory, antibacterial, and other biological properties^6^. Research has also demonstrated that 6-Gingerol can effectively ameliorate cognitive impairment, oxidative stress, neuroplasticity, amyloidosis, and inflammation in a rat model of neuroinflammation induced by lipopolysaccharide (LPS)^7^. Furthermore, 6-Gingerol has been found to inhibit pyroptosis and mitigate sepsis-induced liver injury through activation of the Nrf2 signaling pathway^8^. In addition, 6-Gingerol attenuated I/R injury in heart, brain and small intestine^9–12^, but the role of 6-Gingerol in hepatic I/R injury has not yet been reported.

The activation of the mitogen-activated protein kinase (MAPK) pathway is closely associated with hepatic I/R injury, and inhibition of MAPK signaling pathway activation can significantly reduce hepatic I/R injury^13^. The mitogen activated protein kinase phosphatase (MKP) family acts as a negative regulator of the MAPK signalling pathway by dephosphorylating phosphoserine/threonine and phosphotyrosine residues, which is closely related to many human diseases^14^. MKP5, a member of the MKP family, can inhibit the activation of P38 and JNK to reduce lipotoxicity-induced pancreatic β-cell injury^15^. Previous studies have shown that 6-Gingerol can reduce hypoxia-induced apoptosis in PC-12 cells by inhibiting the P38/JNK signaling pathway^16^. Additionally, in prostate cells, 6-Gingerol has been found to exert anti-inflammatory effects by promoting the expression of MKP5^17^. Based on these findings, we hypothesized that 6-Gingerol could play a protective role in hepatic I/R injury by promoting MKP5 expression and thus inhibiting the P38/JNK signaling pathway.

In this study, we investigated the protective effects of 6-Gingerol on hepatic I/R injury using mouse liver I/R and AML12 cell H/R models. Our results showed that 6-Gingerol could effectively reduce serum ALT and AST levels, attenuate tissue necrosis, decrease hepatocyte apoptosis and inflammatory response after hepatic I/R, and inhibit the phosphorylation of p38/JNK protein. Furthermore, the protective effect of 6-Gingerol was associated with the promotion of MKP5 expression. Therefore, 6-Gingerol has the potential to become a promising drug for the treatment of hepatic I/R injury.

## Materials and methods

### Animals and hepatic I/R injury model

Male C57BL/6 mice (6–8 weeks, 20–25 g) were provided by Zhengzhou University Experimental Animal Center. The mice were housed under pathogen-free conditions with a 12 h of light/darkness cycle, and given ad libitum access to food and water. MKP5 knockout mice were provided by Professor Liang Yinming of Xinxiang Medical University. Information on the construction of MKP5 knockout mice can be found in the supplementary materials. All animal experimental procedures followed the ethical codes of ethics established by the International Animal Committee and the ARRIVE guidelines. The experiment was approved by the Ethics Committee of the First Affiliated Hospital of Zhengzhou University (ethics approval number: 2018-KY-78). 6-Gingerol was dissolved in dimethyl sulfoxide (Solarbio, Beijing, China) and then diluted with normal saline. 6-Gingerol (50 mg/kg and 100 mg/kg) group mice and MKP5 KO groups mice were administered by gavage once a day for five days, and the control group was given equal amounts of saline^8^.

A mouse hepatic I/R injury model was established following the methods reported in the literature^18,19^. Briefly, mice were anesthetized by intraperitoneal injection of 60 mg/kg sodium pentobarbital, a 2 cm incision was made at the mid-abdomen with surgical scissors. The middle and left lobes of the hepatic were separated, and non-invasive arterial clamps were used to clamp the blood vessels of both lobes to simulate ischemia. After 90 min, the clamps were released to restore liver perfusion, and the mid-abdominal incision was closed using silk suture. Mice in the sham-operated group did not undergo clamping and reperfusion operations, and the rest of the operations were the same. Blood and tissue specimens were collected after 6 h of reperfusion for subsequent experimental testing.

### Measurement of serum transaminase levels

After reperfusion, blood samples were collected and centrifuged at 3000 g/min for 5 min, and the supernatant was collected for assay. Serum aspertate aminotransferase (AST) and alanine aminotransferase (ALT) were detected according to the kit instructions (JianChen Bioengineering Institute, Nanjing, China).

### H&E

After reperfusion, liver tissue was obtained, fixed in 10% formalin solution, embedded in paraffin, and cut into 5-μm thick sections. The sections were treated and stained according to the instructions of hematoxylin and eosin reagent (servicebio, Wuhan, China). Briefly, the sections were baked at 60 °C for 2 h, then dewaxed with xylene and soaked in gradient ethanol. The sections were stained with hematoxylin for 5 min, differentiated with hydrochloric ethanol for 30 s, rinsed with water for 15 min, restained with eosin for 2 min, washed with water. The sections were conventionally dehydrated with gradient ethanol, transparent with xylene, and sealed with neutral gum. Photographs were collected under the microscope (Olympus, Tokyo, Japan) and analyzed for histopathological changes in the liver. Statistical analysis of the necrotic area of the liver was performed using Image Pro Plus software (version 6.0), percentage of necrotic area = necrotic area/total area of the field of view.

### Terminal deoxynucleotidyl transferase dUTP nick-end labeling (TUNEL) staining

Paraffin sections were dewaxed, followed by incubation with proteinase K at 37 °C for 20 min. The sections were then washed with PBS and incubated with TUNEL detection solution (servicebio, Wuhan, China) at 37 °C for 10 min. After incubation, the sections were washed three times with PBS for 5 min each, stained with DAPI (servicebio, Wuhan, China), and incubated at room temperature for 10 min. The sections were washed with PBS and sealed with anti-fluorescence quenching solution (servicebio, Wuhan, China) before being photographed under a fluorescent microscope (Olympus, Tokyo, Japan).

### Western blot

Hepatic tissue and cellular proteins were extracted according to the RIPA instructions (Solarbio, Beijing, China), and the proteins concentration were determined using the BCA kit (Solarbio, Beijing, China). 10% or 12% SDS-PAGE were used to separated proteins, then transferred to PVDF membrane, blocked with 5% skim milk powder for 1 h. Primary antibody was added and incubated overnight at 4 °C, followed by incubation with goat anti-rabbit or goat anti-mouse secondary antibody (Proteintech, Wuhan, China) at room temperature for 1 h. The gel imaging system (Cytiva, America) was used for color development after the addition of ECL luminescent solution (NCM Biotech, Suzhou, China). The primary antibodies used for western blot are shown in Table 1. (Original protein bands are provided in the Supplementary file).

**Table 1:** Antibody information for westrn blot.

Antibody	Company	Catalog number	Source	Concentration
Cleaved caspase 3	CST	9664	Rabbit	1:1000
BAX	Proteintech	50599-2-Ig	Rabbit	1:1000
BCL-2	HUABIO	ET1702-53	Rabbit	1:1000
p-p65	CST	3033	Rabbit	1:1000
p65	Proteintech	10745-1-AP	Rabbit	1:1000
p-IKKβ	CST	2697	Rabbit	1:1000
IKKβ	Proteintech	15649-1-AP	Rabbit	1:600
p-IκBα	CST	2859	Rabbit	1:1000
p-p38	CST	4511	Rabbit	1:1000
p38	HUABIO	ET1702-65	Rabbit	1:1000
p-JNK	CST	4668	Rabbit	1:1000
JNK	Proteintech	24164-1-AP	Rabbit	1:1000
MKP5	Santa Cruz	sc-374276	Mouse	1:1000
GAPDH	Proteintech	60004-1-Ig	Mouse	1:5000

### Real-time polymerase chain reaction (PCR) analyses

Total RNA was extracted from liver tissues and cells using a Trizol regent (Solarbio, Beijing, China), and the concentration of total RNA was determined using a micro UV spectrophotometer. Total RNA (1 μg) was reverse transcribed to cDNA using a reverse transcription kit (vazyme,Nanjing, China). cDNA was amplified using SYBR Green qPCR Mix reagent (vazyme,Nanjing, China) on a qPCR instrument. GAPDH was used as an internal reference. 2^−△△CT^ method was used for analysis. The primer sequences used for amplification are shown in Table 2.

**Table 2:** Quantitative polymerase chain reaction primer sequences.

Primer	Primer sequence
IL-1β F	5ʹ-GCTTCAGGCAGGCAGTATCA-3ʹ
IL-1β R	5ʹ-AGTCACAGAGGATGGGCTCT-3ʹ
IL-6 F	5ʹ-AGAGACTTCCATCCAGTTGCC-3ʹ
IL-6 R	5ʹ-TCCTCTGTGAAGTCTCCTCTCC-3ʹ
TNF-α F	5ʹ-AGCCGATGGGTTGTACCTTG-3ʹ
TNF-α R	5ʹ-ATAGCAAATCGGCTGACGGT-3ʹ
MCP-1 F	5ʹ-ATCTGCCCTAAGGTCTTCAGC-3ʹ
MCP-1 R	5ʹ-AGGCATCACAGTCCGAGTCA-3ʹ
GAPDH F	5ʹ-CTGCCCAGAACATCATCCCT-3ʹ
GAPDH R	5ʹ-TACTTGGCAGGTTTCTCCAGG-3ʹ

### Immunohistochemical staining

Paraffin sections were baked at 60 °C for 2 h, dewaxed by xylene immersion, dehydrated in gradient ethanol, and sections were subjected to thermal antigen repair with 0.01 mol/L citrate buffer (pH 6.0) and incubated with 3% H_2_O_2_ at room temperature for 10 min to inactivate endogenous enzymes. The sections were incubated with 10% goat serum for 1 h at room temperature, and incubated with Ly6g primary antibody (1:200, servicebio, Wuhan, China) overnight at 4 °C. On the second day, secondary antibody (1:500, servicebio, Wuhan, China) was added and incubated at room temperature for 30 min. The sections were washed with PBS three times before and after the secondary antibody incubation. DAB chromogenic solution was added dropwise, followed by restaining with hematoxylin and sealing with neutral gum. Microscopic examination was performed and images were collected.

### Cell culture and H/R model

AML12 mouse hepatocytes were purchased from Procell Biotechnology Co., Ltd. Cells were cultured in DMEM/F12 medium (Procell, Wuhan, China) containing 10% fetal bovine serum (Gibco, Carlsbad,CA, USA), 1 × 10^5^ U/L penicillin and 100 mg/L streptomycin (Solarbio, Beijing, China) in a constant temperature incubator at 37 °C with 5% CO_2_. The H/R model of AML12 cells was established according to reference^20^. When the cell density reached 70%-80%, the medium was discarded, and the cells were washed twice with PBS, sugar-free and serum-free medium (Procell, Wuhan, China) was added, and the cells were placed in a triple-vapor incubator (5% CO_2_, 94% N_2_ and 1% O_2_) for hypoxia. After 12 h, the medium was changed to normal medium, and the cells were placed in a normoxic incubator for another 6 h to complete reoxygenation.

### Cell counting kit-8 assay

AML12 cells were inoculated in 96-well plates (100 μL/well) at a density of 0.5 × 10^4^ cells/mL and incubated in an incubator at 37 °C with 5% CO_2_. When the cell density reached 70–80%, H/R was performed according to the experimental groups. After reoxygenation, 10 μL of CCK-8 reagent (Beyotime, Shanghai, China) was added to each well, and the cells were incubated in the incubator for 1h, and the OD value at 450 nm was measured using an enzyme marker (Thermo Fisher Scientific, Inc.), and the cell activity was calculated according to the absorbance value.

### Flow cytometry analysis

After reoxygenation, cells were collected by trypsin digestion, centrifuged at 200 g/min for 5 min, washed twice with PBS, and resuspended in 195 μL of 1× Annexin-binding buffer. The cells were then incubated with 5 μL of Annexin V-FITC and 10 μL of PI for 15 min in the dark. Cell apoptosis rates were analyzed using flow cytometry (BD Biosciences, USA).

### Statistical analysis

The experimental data were analyzed using SPSS 2 2.0 statistical software. The measurement data were expressed as mean ± standard deviation. The t-test was used for comparisons between two groups. One-way ANOVA was used for comparisons between multiple groups. The difference was statistically significant at *P* < 0.0 5.

## Results

### 6-Gingerol alleviates hepatic damage during liver I/R injury

**Figure 1:**
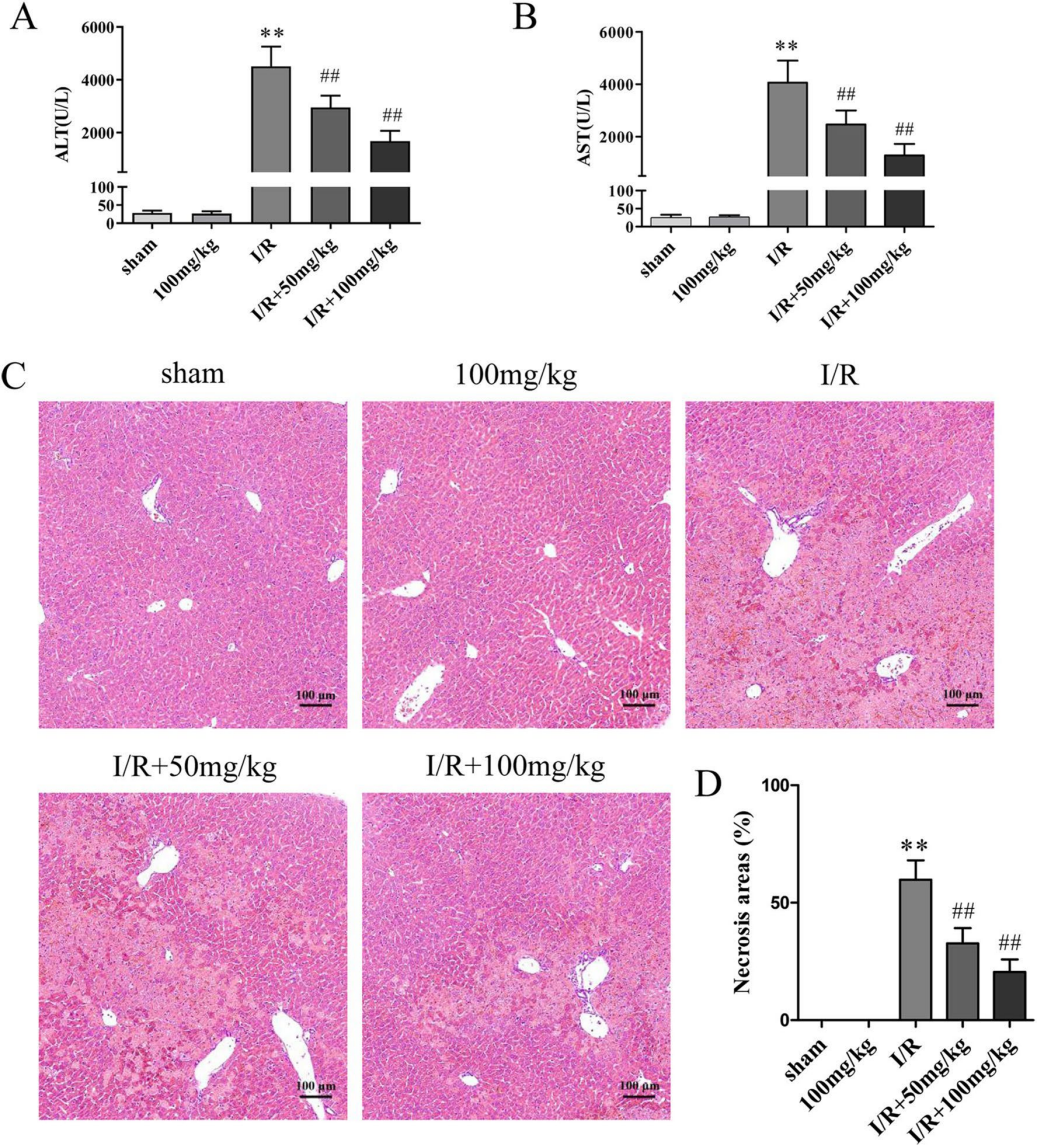
6-Gingerol alleviates hepatic damage during liver IR injury. (**A**)Serum ALT and (**B**) AST level, (**C**) H&E staining and (**D**) necrotic area statistics of liver tissue in mice. ^**^P < 0.01 versus sham group; ^##^*P* < 0.01 versus I/R group.

Serum ALT and AST levels were significantly higher in the I/R group compared to the sham-operated group (Fig. 1A,B). However, the I/R+6-Gingerol (50 mg/kg, 100 mg/kg) group showed significantly lower levels compared to the I/R group (Fig. 1A,B). H&E staining revealed that the hepatocytes in the I/R group exhibited focal or large areas of degenerative necrosis, with disrupted liver lobule structure (Fig. 1C). In contrast, the area of cell necrosis in the I/R+6-Gingerol (50 mg/kg, 100 mg/kg) group was significantly reduced compared to the I/R group, and the liver tissue structure was largely intact (Fig. 1C,D). These results suggest that 6-Gingerol plays a protective role against I/R injury in mouse liver.

### 6-Gingerol alleviates apoptosis in hepatic I/R injury

**Figure 2:**
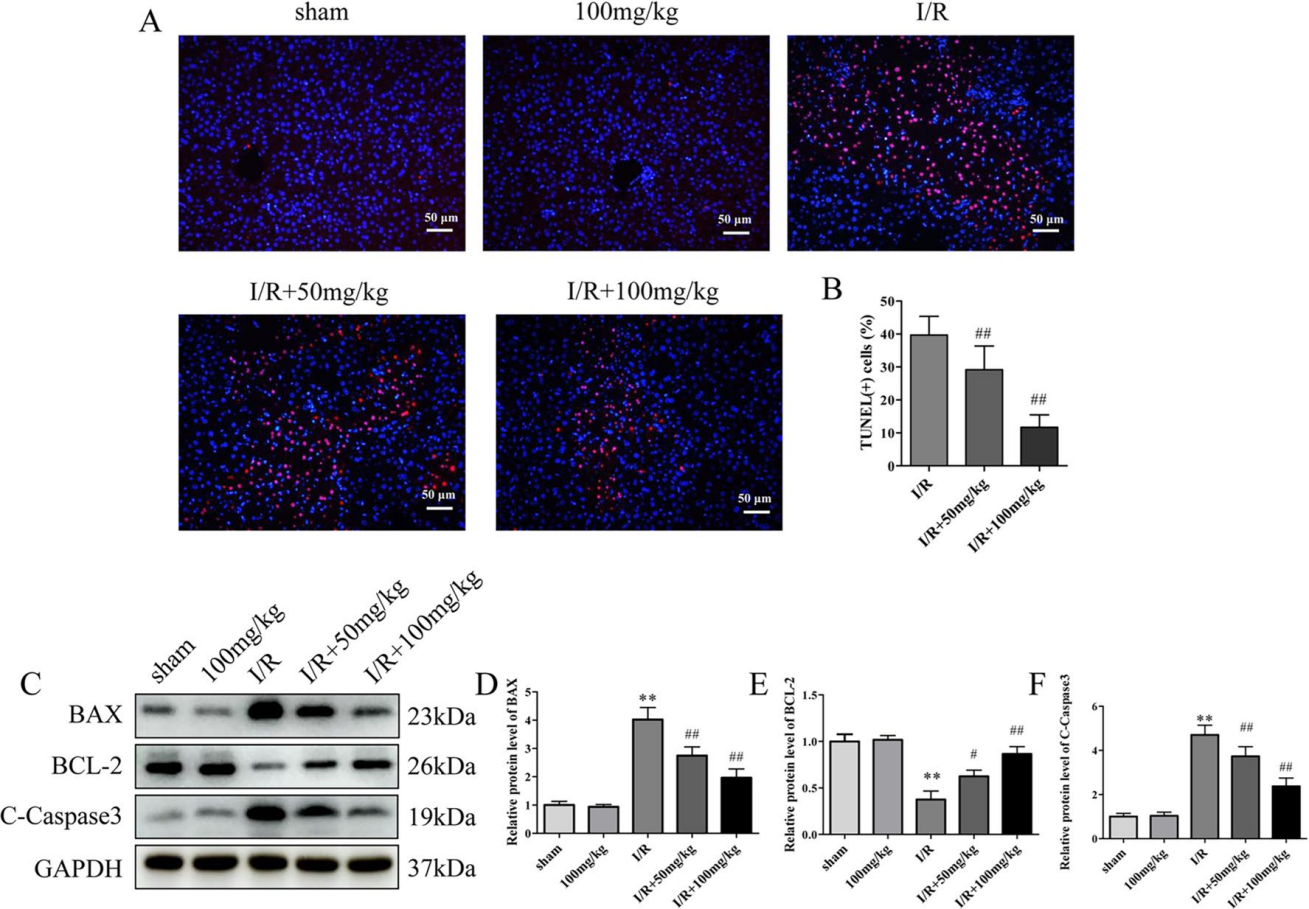
6-Gingerol alleviates apoptosis in hepatic I/R injury. (**A**) TUNEL staining (red fluorescence indicates TUNEL positives cells) and (**B**) statistical analysis of mouse liver tissue, (**C**) detection of apoptotic proteins and (**D**–**F**) statistical analysis in mouse liver tissue. ^**^P < 0.01 versus sham group; ^##^*P* < 0.01 versus I/R group.

The number of TUNEL-positive cells and the protein expression of BAX, C- caspase3 were significantly increased, while protein expression of BCL-2 was significantly decreased in hepatic tissues of mice after I/R injury (Fig. 2A–F). In contrast, 6-Gingerol pretreatment significantly decreased the protein of BAX, C-caspase3 as well as the number of TUNEL-positive cells in liver tissue after hepatic I/R, and increased the protein expression of BCL-2 (Fig. 2A–F). These results suggest that 6-Gingerol pretreatment restrained the activation of apoptotic pathways in mouse hepatic after I/R injury.

### 6-Gingerol alleviates inflammation in hepatic I/R injury

**Figure 3:**
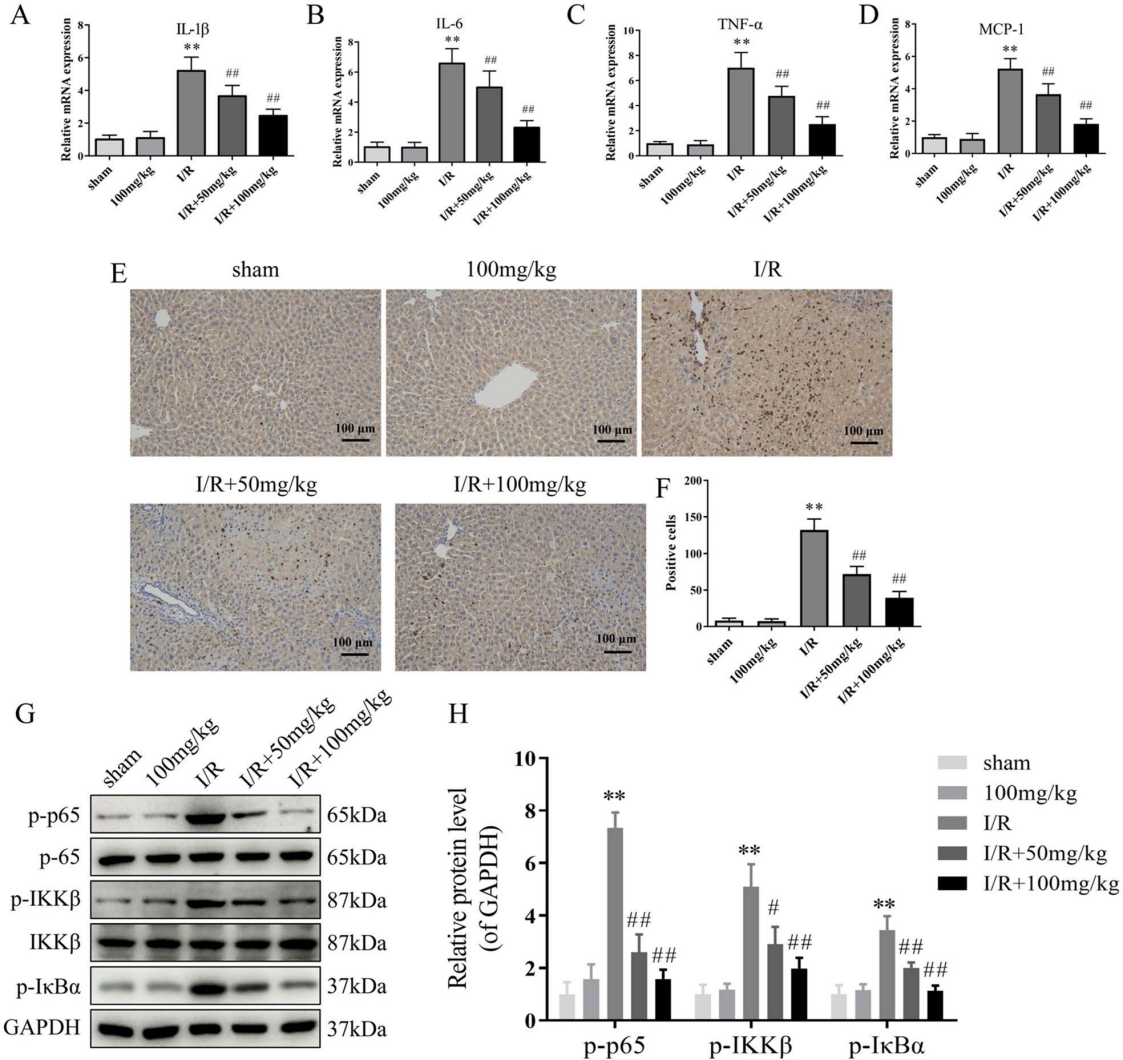
6-Gingerol alleviates inflammation in hepatic I/R injury. (**A**–**D**) mRNA expression of inflammatory cytokines *Il-1β, Il-6, Tnf-α and MCP-1,* (**E**) Ly6g immunohistochemical staining (brown stained cells indicate Ly6g positive) and (**F**) statistical analysis in mouse liver tissues, (**G**) Western blotting was used to detect NF-κB signaling pathway proteins and (**H**) statistical analysis ^**^P < 0.01 versus sham group; ^#^*P* < 0.05 and ^##^*P* < 0.01 versus I/R group.

The mRNA expression of inflammatory factors *Il-1β, Il-6, Tnf-α* and *MCP-1* was significantly increased in mouse liver tissues after I/R (Fig. 3A–D), while 6-Gingerol pretreatment significantly decreased the mRNA expression of these inflammatory factors in liver tissues after I/R (Fig. 3A–D). Moreover, immunohistochemical results showed that the number of Ly6g-positive cells was significantly increased in liver tissues after I/R, while the number of Ly6g-positive cells was significantly decreased in the IR+6-Gingerol (50 mg/kg, 100 mg/kg) group (Fig. 3E,F). Additionally, western blotting results showed that 6-Gingerol pretreatment inhibited the activation of the NF-κB signaling pathway after hepatic I/R, which was manifested as reduced phosphorylation of p65, IKKβ and IκBα proteins (Fig. 3G,H). These results suggest that 6-Gingerol reduces the tissue inflammatory response after I/R injury in mice.

### 6-Gingerol alleviates cell injury induced by H/R

**Figure 4:**
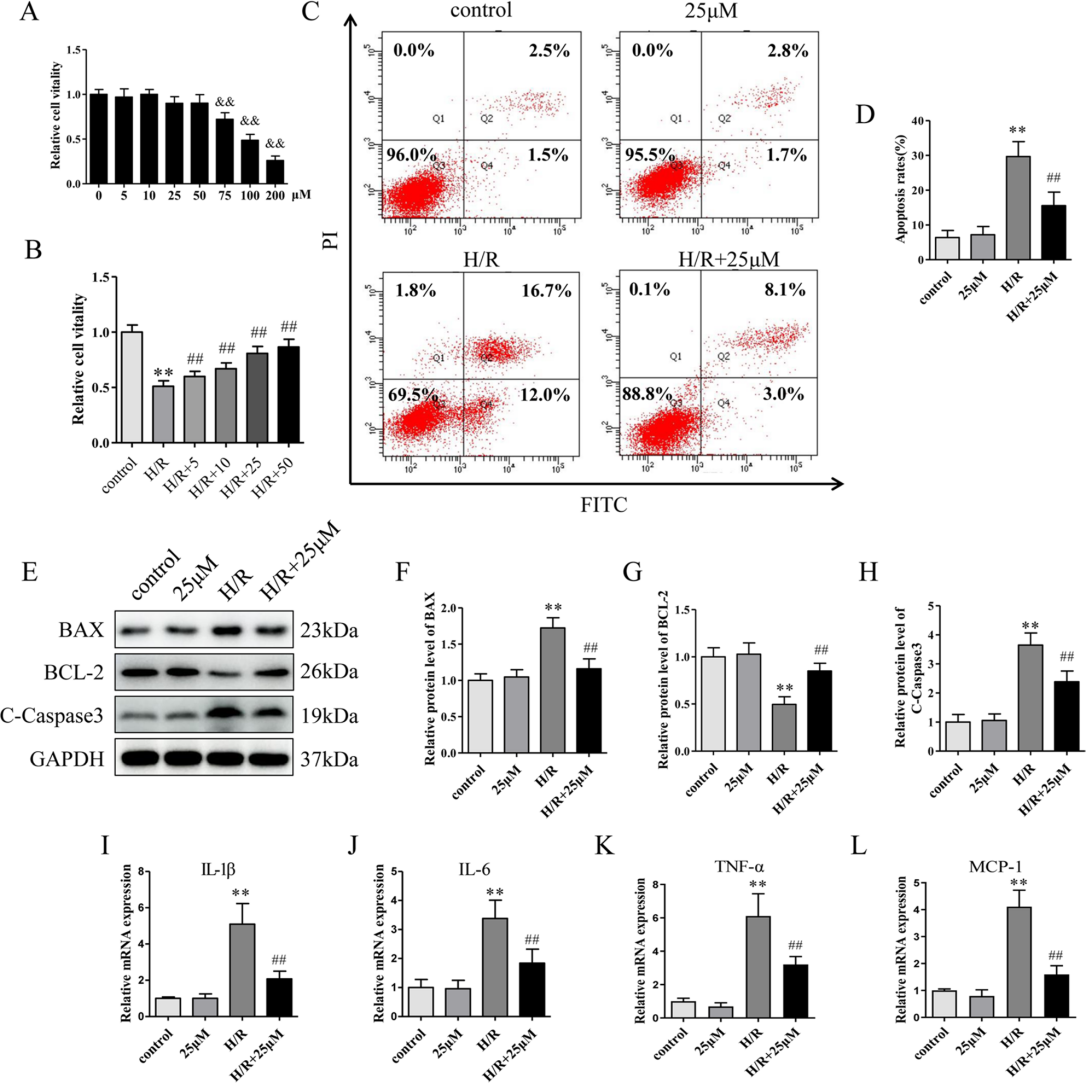
6-Gingerol alleviates cell injury induced by H/R. (**A**) Effect different concentrations of 6-Gingerol on the activity of AML12 cells under normal oxygen conditions, (**B**) effects of different concentrations of 6-Gingerol on cell viability after H/R. (**C**, **D**) Cell apoptosis was determined by flow cytometry. (**E**) Western blotting was used to detect apoptosis-related proteins and (**F**–**H**) statistical analysis, (**I**–**L**) mRNA expression of inflammatory cytokines *Il-1β, Il-6, Tnf-α and MCP-1.* ^**^P < 0.01 versus control group; ^#^*P* < 0.05 and ^##^*P* < 0.01 versus H/R group.

To determine the concentration of 6-gingerol used in cellular experiments, the CCK-8 assay was used to detect cell activity after 24 h pretreatment with different concentrations (5, 10, 25, 50, 75, 100 and 200 µM) of 6-gingerol. The results showed that 6-gingerol produced cytotoxic effects when the drug concentration exceeded 50 µM (Fig. 4A), thus the concentration of 6-gingerol was kept below 50 μM in cell H/R experiments. We then examined the protective effect of different concentrations of 6-gingerol on H/R-induced cell damage in AML12 cells. Cell activity was significantly reduced after H/R compared to the control group, but pretreatment with 6-gingerol (5, 10, 25, and 50 µM) increased cell activity compared to the H/R group, and there was no difference in the increase of cell activity between 25 and 50 µM 6-gingerol (Fig. 4B), so the final experimental concentration of 6-gingerol was 25 µM. H/R injury significantly increased the apoptosis rates (Fig. 4C,D) and the expressions of pro-apoptotic proteins BAX and C-Caspase3 (Fig. 4E–H), while inhibited the expression of anti-apoptotic protein BCL-2 (Fig. 4E–H). 6-Gingerol pretreatment inhibited H/R-induced apoptosis, reversed the alteration of apoptosis-related proteins (Fig. 4E–H). Moreover, 6-gingerol pretreatment significantly decreased the mRNA expression of inflammatory factors *Il-1β, Il-6, Tnfα* and *MCP-1* after H/R in AML12 cells (Fig. 4I–L). These results suggest that 6-Gingerol alleviates H/R-induced cell damage.

### 6-Gingerol inhibits P38/JNK signaling during hepatic I/R injury in vivo and in vitro

**Figure 5:**
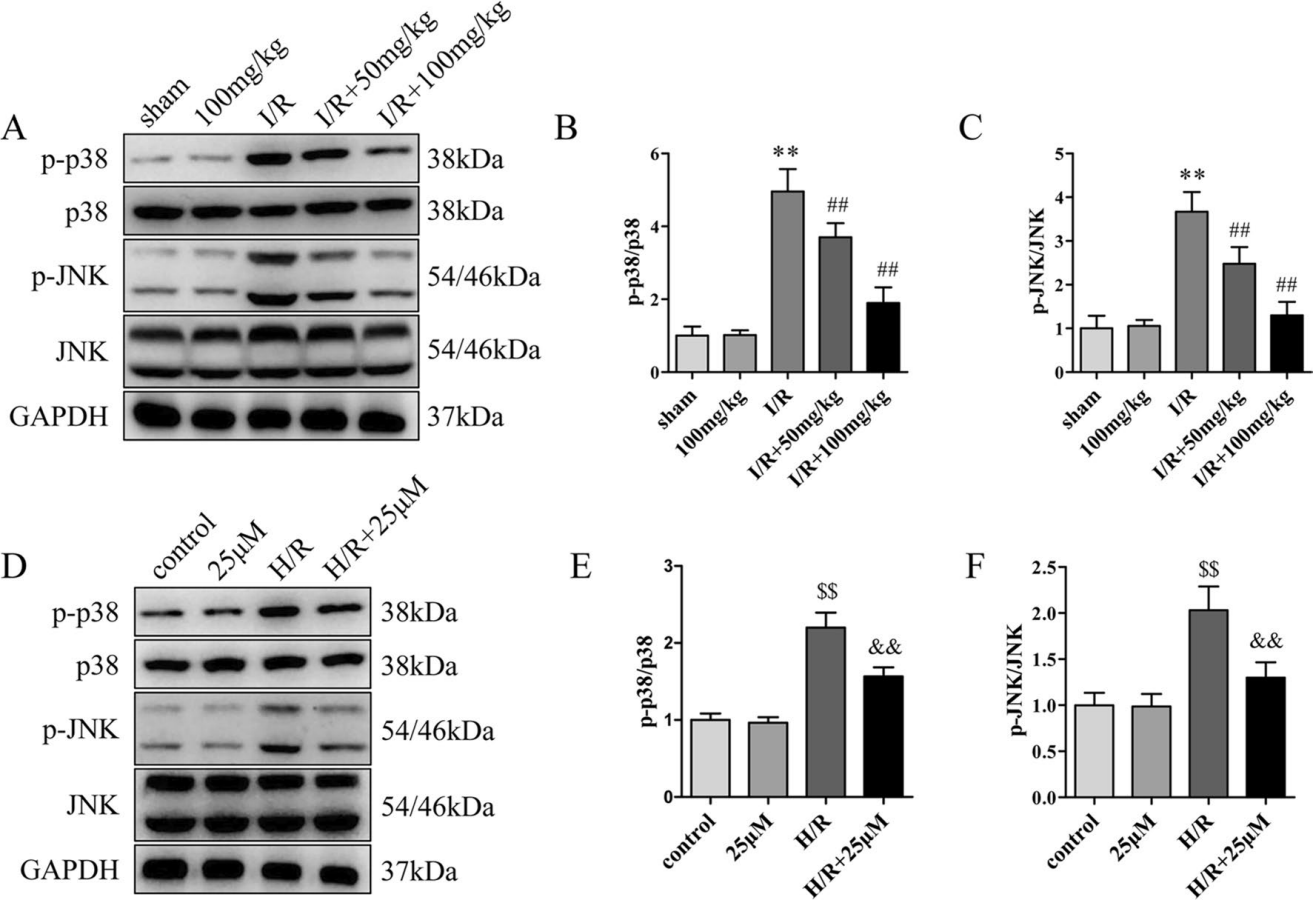
6-Gingerol inhibits P38/JNK signaling during hepatic I/R injury in vivo and in vitro. (**A**) Proteins detection and (**B**, **C**) statistical analysis in mouse liver tissue, (**D**) proteins detection and (**E**, **F**) statistical analysis in AML12 cells. ^**^P < 0.01 versus sham group; ^##^*P* < 0.01 versus I/R group; ^$$^*P* < 0.01 versus control group; ^&&^*P* < 0.01 versus H/R group.

The P38/JNK signaling is involved in the regulation of hepatic I/R injury. To detect changes in the P38/JNK signaling pathway, we examined the changes in P38/JNK protein phosphorylation in mice and cells. Compared with the control group, protein phosphorylation of P38 and JNK was significantly increased in liver tissue after I/R injury (Fig. 5A–C) and AML12 cells subjected to H/R injury (Fig. 5D–F). And 6-Gingerol pretreatment significantly reduced the protein phosphorylation of P38 and JNK after I/R and H/R injury (Fig. 5A–F). These results suggest that 6-Gingerol pretreatment inhibits the activation of P38/JNK signaling pathway after hepatic I/R in mice.

### 6-Gingerol promoted the expression of MKP5 in vivo and in vitro

**Figure 6:**
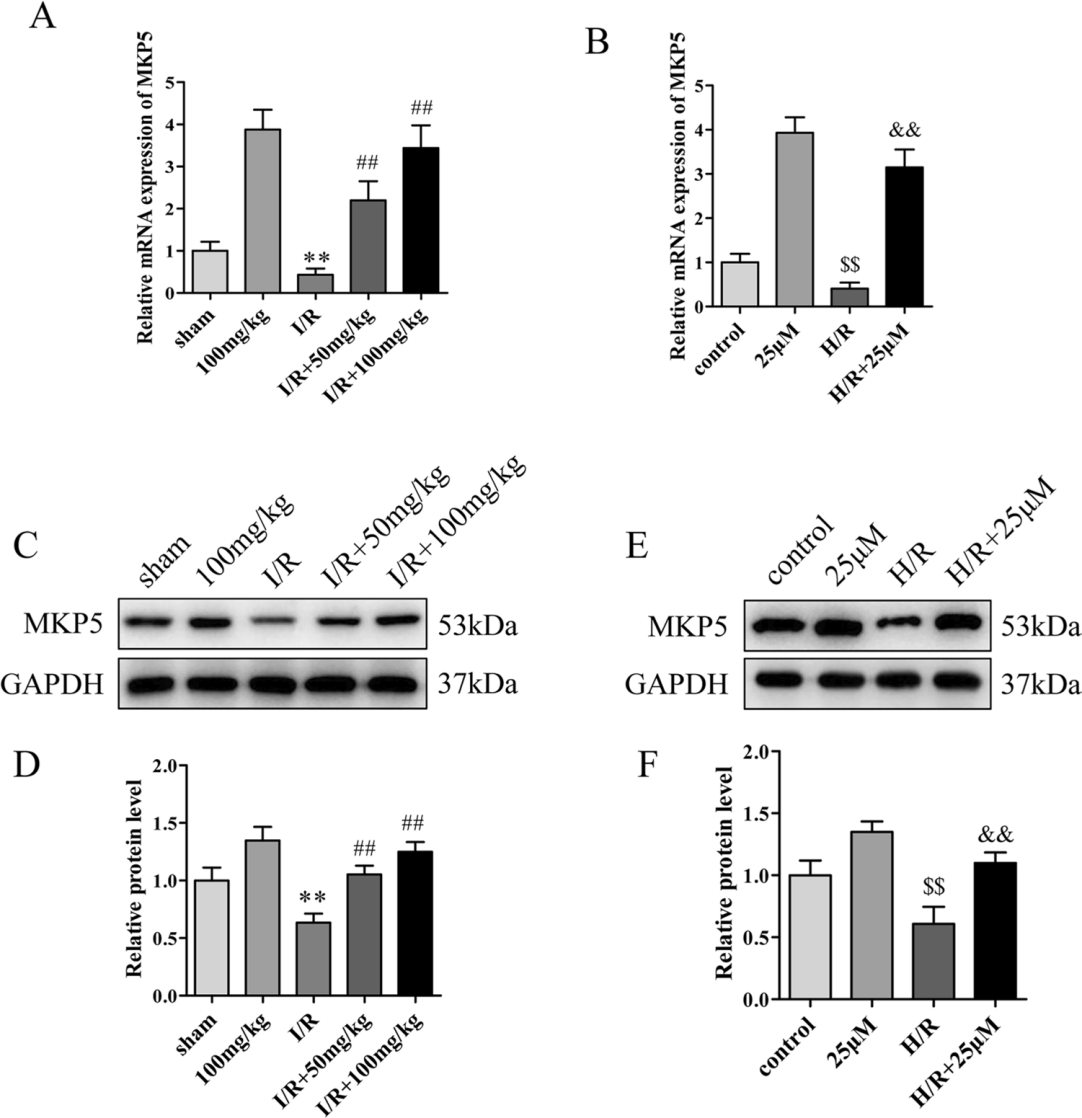
6-Gingerol promoted the expression of MKP5 in vivo and in vitro. (**A**) mRNA expression of MKP5 in liver tissues, (**B**) mRNA expression of MKP5 in AML12 cells, (**C**) protein detection and (**D**) statistical analysis of MKP5 in liver tissues, (**E**) protein detection and (**F**) statistical analysis of MKP5 in liver AML12 cells. ^**^P < 0.01 versus sham group; ^##^*P* < 0.01 versus I/R group; ^$$^*P* < 0.01 versus control group; ^&&^*P* < 0.01 versus H/R group.

Previous study has also shown that 6-Gingerol could promote MKP5 expression in prostate cells^17^, and our previous studies have shown that MKP5 is involved in the regulation of hepatic I/R injury by inhibiting the P38/JNK signaling pathway^21^. We verified by molecular docking that 6-gingerol was able to bind to the MKP5 protein (Results can be found in the supplementary materials). Then, we examined the effect of 6-Gingerol on MKP5 mRNA and protein expression after hepatic I/R injury. As shown in Fig. 6A,B, MKP5 mRNA expression was significantly decreased after I/R and H/R injury, and 6-Gingerol could promote MKP5 mRNA expression after I/R and H/R injury. We further examined the changes in MKP5 protein expression, and the results showed that MKP5 protein expression was significantly increased after 6-Gingerol pretreatment compared with sham group mice and control cells, and also increased the I/R and H/R-induced decrease in MKP5 protein expression (Fig. 6C–F). These results suggest that 6-Gingerol can promote MKP5 expression.

### Knockout of MKP5 attenuated the hepatoprotective effect of 6-Gingerol

**Figure 7:**
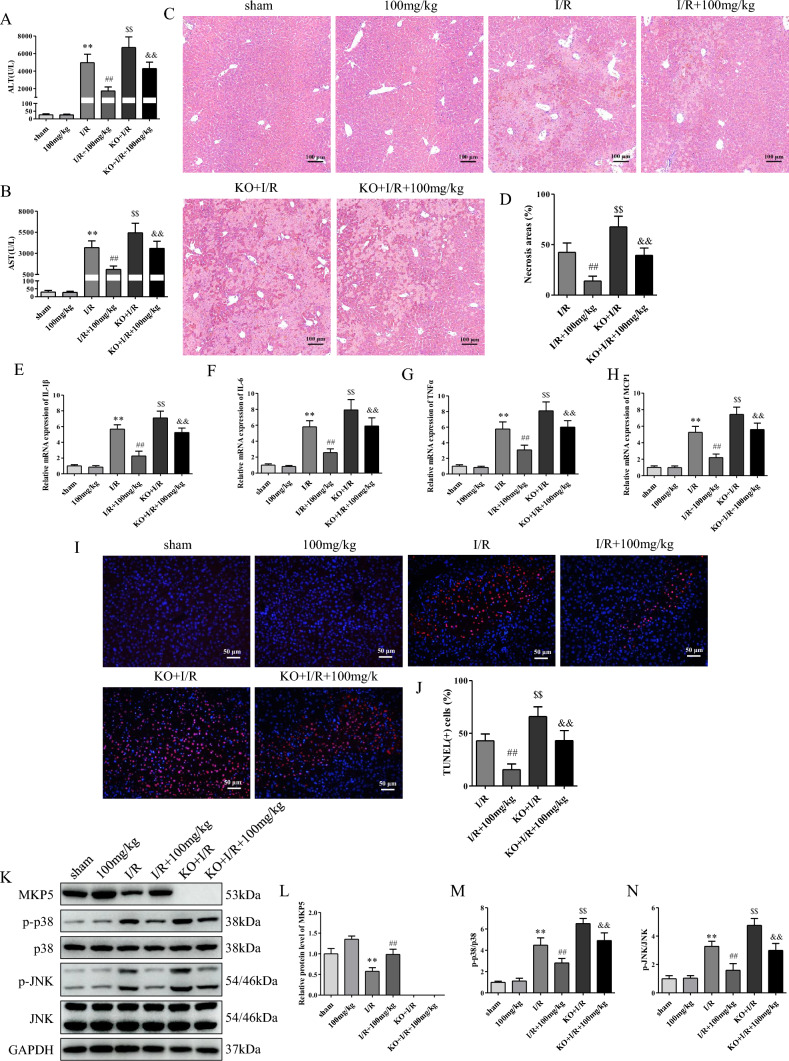
Knockout of MKP5 attenuated the hepatoprotective effect of 6-Gingerol. (**A**, **B**) Serum ALT and AST level, (**C**) H&E staining and (**D**) necrotic area statistics, (**E**–**H**) *Il-1β, Il-6, Tnf-α* and* Mcp1* mRNA expression, (**I**) TUNEL staining (red fluorescence indicates TUNEL positives cells) and (**J**) statistical analysis, (**K**–**N**) MKP5 protein and P38/JNK pathway protein detection. ^**^P < 0.01 versus sham group; ^##^*P* < 0.01 versus I/R group; ^$$^*P* < 0.01 versus I/R group; ^##^*P* < 0.01 versus I/R +100 mg/kg group.

To further investigate whether 6-Gingerol protects against hepatic I/R injury depends on MKP5, we performed a mechanistic investigation using MKP5 KO mouse. Compared with WT mice, the ALT and AST levels (Fig. 7A,B), liver necrosis area (Fig. 7C,D), inflammatory response (Fig. 7E–H), and hepatocyte apoptosis (Fig. 7I,J) were significantly increased, and P38/JNK protein phosphorylation was more pronounced after MKP5 KO (Fig. 7K–N). The protective effects and inhibition of P38/JNK protein phosphorylation by 6-Gingerol were significantly diminished in MKP5 KO mice compared with WT mice (Fig. 7A–N), and these results suggest that MKP5 KO attenuated the protective effects of 6-Gingerol and that the hepatoprotective effects of 6-Gingerol were associated with modulation of MKP5 expression.

## Discussion

Apoptosis and inflammatory response are key causes of liver I/R injury and important targets to mitigate hepatic I/R injury^22^. In this study, we established a mouse hepatic I/R injury model and AML12 cells H/R injury model to investigate the potential effects of 6-Gingerol on hepatic injury. The results showed that 6-Gingerol inhibited liver injury, apoptosis and inflammation, increased cellular activity, and inhibited JNK/P38 protein phosphorylation after liver I/R or cellular H/R injury. The protective effect of 6-Gingerol was associated with the promotion of MKP5 expression, and the protective effects and inhibition of p38/JNK pathway of 6-Gingerol was significantly diminished after MKP5 KO. Thus, our results suggest that 6-Gingerol exerts anti-inflammatory and anti-apoptotic effects to attenuate hepatic I/R injury by regulating MKP5-mediated P38/JNK signaling pathway.

Studies have shown that hepatic I/R injury manifests as an increase in inflammatory factors and apoptosis, and that protection against I/R-induced hepatic injury may be achieved by inhibiting inflammatory or apoptosis-related signaling pathways. Han et al. reported that 6-Gingerol attenuated cardiotoxicity caused by arsenic trioxide by inhibiting apoptotic and inflammatory responses^23^. Luo et al. showed that 6-Gingerol exerted a protective effect against cerebral I/R injury by inhibiting NLRP3 inflammatory vesicles and apoptosis^10^. These studies suggest that 6-Gingerol has potent anti-inflammatory and anti-apoptotic effects. In the present study, we examined the mRNA expression of inflammatory factors *Il-1β, Il-6, Tnf-α*, *MCP-1,* and demonstrated that 6-Gingerol pretreatment significantly reduced the expression of these inflammatory factors. Furthermore, immunohistochemical staining showed that 6-Gingerol pretreatment significantly reduced the infiltration of Ly6g-positive cells. The detection of NF-κB pathway protein also proved the anti-inflammatory effect of 6-Gingerol after hepatic I/R injury. And TUNEL staining showed that 6-Gingerol pretreatment significantly reduced apoptosis. Apoptosis-associated protein further verified the anti-apoptotic effect of 6-Gingerol. The results of in vitro AML12 cells H/R test were consistent with those of in vivo I/R test. Thus our results suggest that 6-Gingerol can attenuate hepatic I/R injury by inhibiting the inflammatory response and apoptosis.

The MAPK signaling pathway is an important pathway for the transduction of membrane receptor signals into cells and is involved in the regulation of cell growth, differentiation, migration, and inflammation^24^. MAPK family includes the extracellular signal-regulated kinase (ERK) pathway, the c-Jun N-terminal kinase (JNK) pathway, and the P38 MAPK pathway^25^. The MAPK family plays a critical role in mediating hepatic I/R injury, regulating hepatocyte death and survival by mediating signaling pathways related to apoptosis and inflammatory responses. For example, Regulator of G-protein signaling 14 attenuates hepatic I/R injury by inhibiting the TAK1-mediated JNK/p38 pathways signaling pathway^26^. Ring finger protein 5 protects against hepatic I/R injury by inhibiting the activity of ASK1-JNK/p38 pathway^27^. 6-Gingerol has been reported to inhibit the secretion of pro-inflammatory cytokines by targeting the MAPK signaling pathway, thereby comprehensively inhibiting the development of sepsis^28^. Similarly, in our study, 6-Gingerol inhibited the JNK/p38 pathway in mouse hepatic I/R injury and hepatocyte H/R injury, indicating that 6-Gingerol may protect against hepatic I/R injury by regulating the P38/JNK signaling pathway.

MKP5 belongs to the MKP family, which inactivates target kinase activity by dephosphorylating phosphoserine/threonine and phosphotyrosine residues, acting as a negative regulatory element that regulates the MAPK signaling pathway, which is closely associated with a variety of human diseases^29,30^. Studies have shown that MKP5 can inhibit the activation of P38/JNK signaling pathway to attenuate lipotoxicity-induced pancreatic β-cell injury^15^. Conversely, MKP5 KO in mice aggravates LPS-induced lung injury by enhancing the activation of the P38/JNK signaling pathway^31^. Research has also shown that 6-Gingerol could promote MKP5 expression in prostate cells, exerting anti-inflammatory effects^17^. Our studies shown that 6-Gingerol could promote mRNA and protein expression of MKP5 in the hepatic and AML12 cells. To explore whether the hepatoprotective effect of 6-Gingerol is related to the promotion of MKP5 expression, we verified it using MKP5 KO mice. Consistent with our previous research, the activation of P38/JNK signaling pathway was more pronounced and liver damage was more severe after MKP5 KO^21^, and the protective effect of 6-Gingerol was significantly diminished in MKP5 KO mice. These results suggest that the hepatoprotective effect of 6-Gingerol is dependent on MKP5-mediated P38/JNK signaling pathway.

To summarize, the results of this study suggest that 6-Gingerol has potential as a therapeutic agent for attenuating hepatic I/R injury by inhibiting inflammation and apoptosis. The protective mechanism may involve the promotion of MKP5 expression, which inhibits the activation of the JNK/P38 signaling pathway. However, this study also has some limitations. the present study focused on investigating the effects of 6-Gingerol on apoptosis and inflammatory responses via the P38/JNK signaling pathway in hepatic I/R injury, while whether 6-Gingerol can ameliorate hepatic I/R injury by regulating other signaling pathways remains to be further investigated, and the regulatory relationship between 6-gingerol and MKP5 needs further study. Moreover, we used MKP5 global knockout mice to investigate the mechanism of 6-Gingerol on hepatic I/R injury, and the results would be more convincing if liver-specific knockout mice were used.

## Supplementary Information

Supplementary Figures.

Supplementary Information 2.

## Data Availability

The data that support the findings of this study are available from the corresponding author on reasonable request.

## Abbreviations

I/R: Ischemia reperfusion
H/R: Hypoxia/reoxygenation
ALT: Alanine aminotransferase
AST: Aspartate aminotransferase
MKP5: Mitogen activated protein kinase phosphatase 5
MAPK: Mitogen-activated protein kinase
JNK: c-Jun N-terminal kinase
KO: Knockout
ERK: Extracellular signal-regulated kinase
TUNEL: Terminal-deoxynucleoitidyl transferase mediated nick end labeling
DHE: Dihydroethidium
Bax: Bcl2-associated x protein
Bcl2: B-cell leukemia/lymphoma
PVDF: Polyvinylidene fluoride
BCA: Bicinchoninic acid
CCK-8: Cell counting kit-8
PBS: Phosphate-buffered saline
GAPDH: Glyceraldehyde 3-phosphate dehydrogenase
ANOVA: Analysis of variance
