# Correction: 6-Gingerol attenuates hepatic ischemia/reperfusion injury through regulating MKP5-mediated P38/JNK pathway

**DOI:** 10.1038/s41598-026-61604-5

**Published:** 2026-07-15

**Authors:** Qiwen Yu, Jiye Li, Mengwei Cui, Chaopeng Mei, Qianqian He, Xiaoxiao Du

**Affiliations:** 1 Department of Emergency Medicine, The First Affiliated Hospital of Zhengzhou University, Zhengzhou, 450052 China; 2 Department of Hepatobiliary and Pancreatic Surgery, The First Affiliated Hospital of Zhengzhou University, 1 Jianshe East Road, Erqi, Zhengzhou, 450052 Henan China

Correction to: *Scientific Reports* 10.1038/s41598-024-58392-1, published online 02 April 2024

The original version of this Article contained an error in Fig. 7, where the image displayed in the panel ‘I/R’ in Fig. 7I originated from the image of Fig. 2A panel ‘IR + 100 mg/kg’. As a result, the correct image for the Fig. 7I ‘I/R’ was omitted during figure preparation. The original Fig. 7 and accompanying legend appear below.

**Fig. 7:**
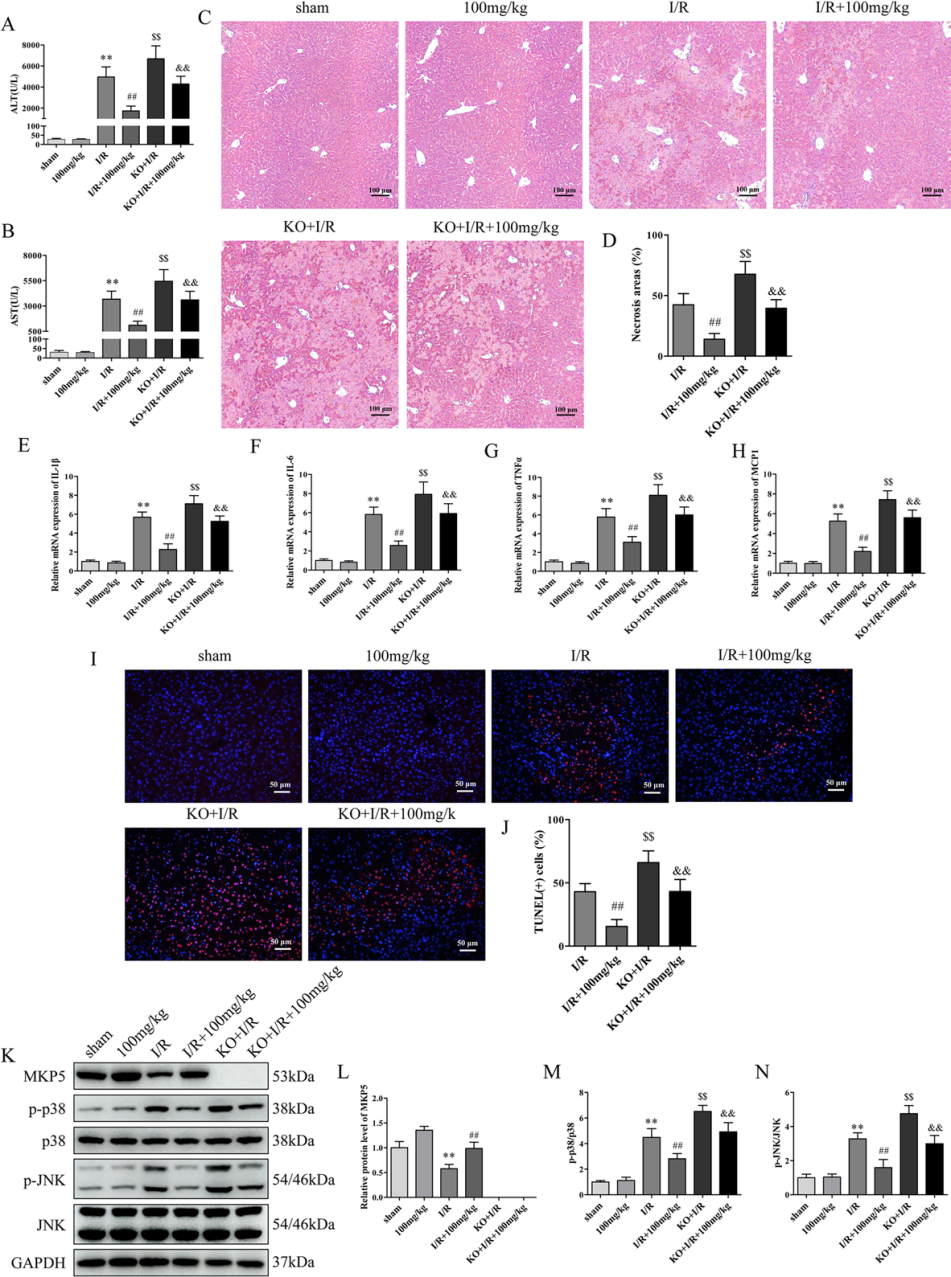
Knockout of MKP5 attenuated the hepatoprotective effect of 6-Gingerol. (**A**, **B**) Serum ALT and AST level, (**C**) H&E staining and (**D**) necrotic area statistics, (**E**–**H**) *Il-1β, Il-6, Tnf-α* and *Mcp1* mRNA expression, (**I**) TUNEL staining (red fluorescence indicates TUNEL positives cells) and (**J**) statistical analysis, (**K**–**N**) MKP5 protein and P38/JNK pathway protein detection. ^**^P < 0.01 versus sham group; ^##^*P* < 0.01 versus I/R group; ^$$^*P* < 0.01 versus I/R group; ^##^*P* < 0.01 versus I/R + 100 mg/kg group.

The original Article has been corrected.

