# Supplementary material for: 6-Gingerol attenuates hepatic ischemia/reperfusion injury through regulating MKP5-mediated P38/JNK pathway
Source: Sci Rep. 2024 Apr 2;14:7747. doi: 10.1038/s41598-024-58392-1 (PMC10987508; doi:10.1038/s41598-024-58392-1)
Supplement: Supplementary file 1 — Supplementary Figures. [file 41598_2024_58392_MOESM1_ESM.pdf]

Figure S1

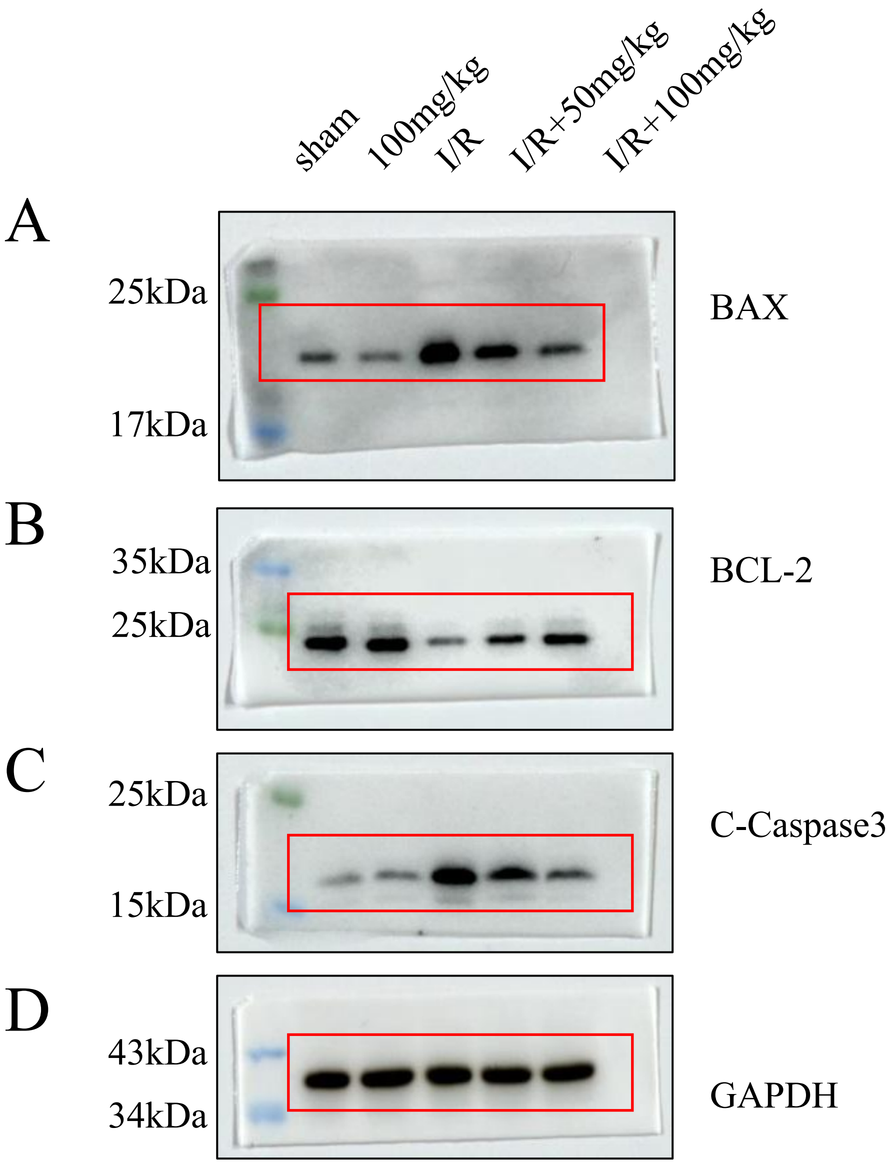

(A) BAX protein bands in Figure 2C; (B) BCL-2 protein bands in Figure 2C;
(C) C-Caspase3 protein bands in Figure 2C; (D) GAPDH protein bands in Figure 2C.

Figure S2

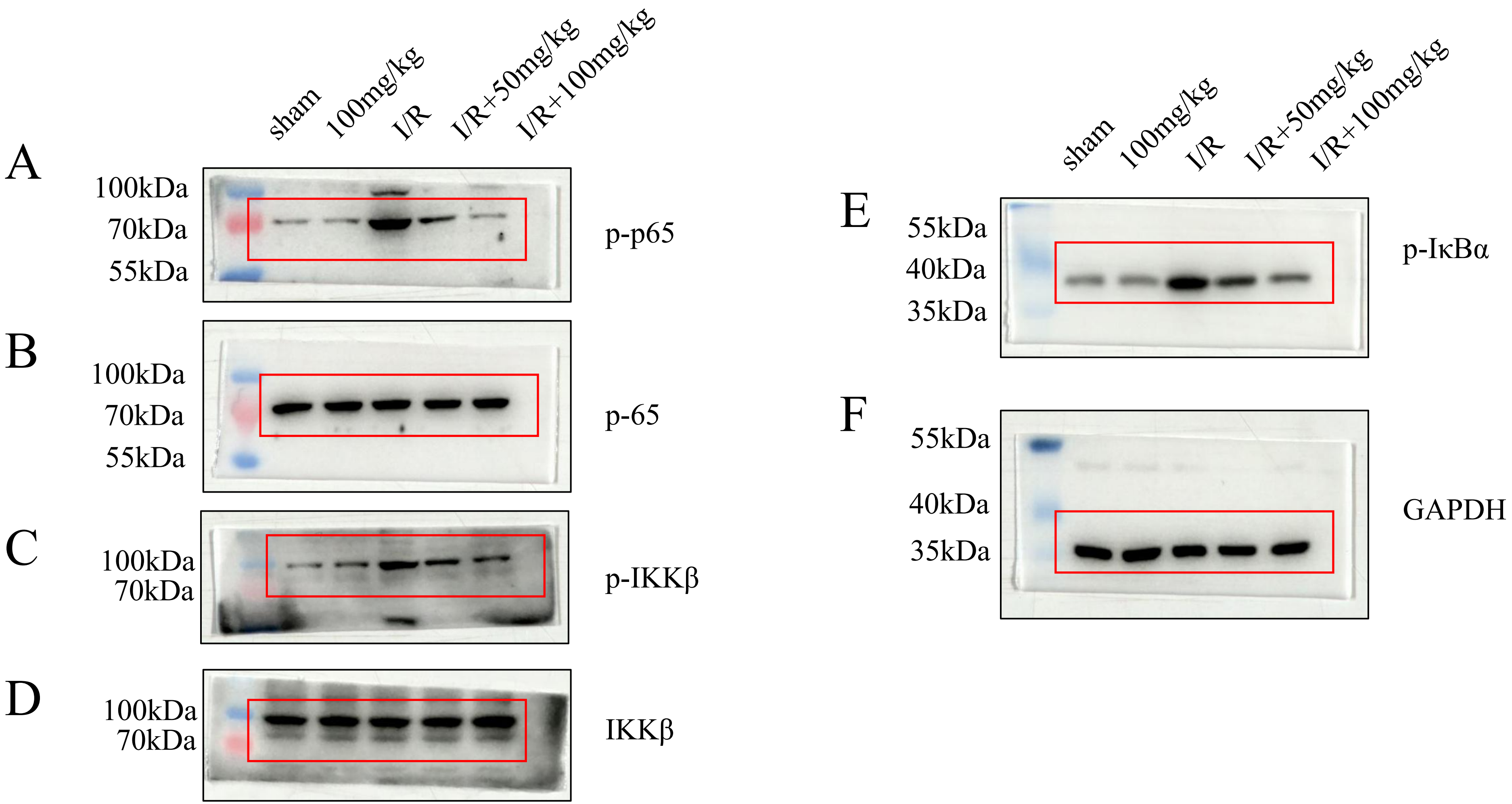

(A) p-p65 protein bands in Figure 3G; (B) p-65 protein bands in Figure 3G; (C) p-IKKβ protein bands in Figure 3G; (D) IKKβ protein bands in Figure 3G; (E) p-IκBα protein bands in Figure 3G; (F) GAPDH protein bands in Figure 3G .

Figure S3

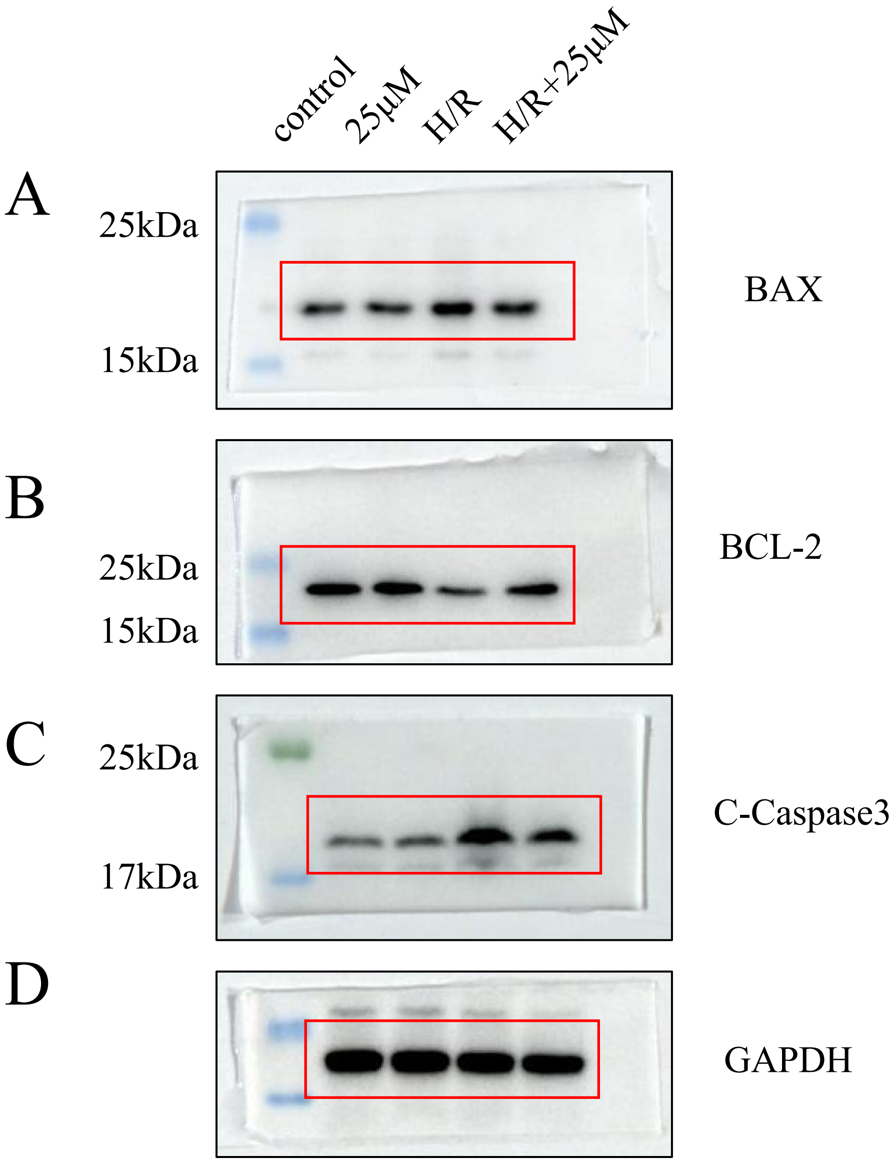

(A) BAX protein bands in Figure 4E; (B) BCL-2 protein bands in Figure 4E;
(C) C-Caspase3 protein bands in Figure 4E; (D) GAPDH protein bands in Figure 4E.

Figure S4

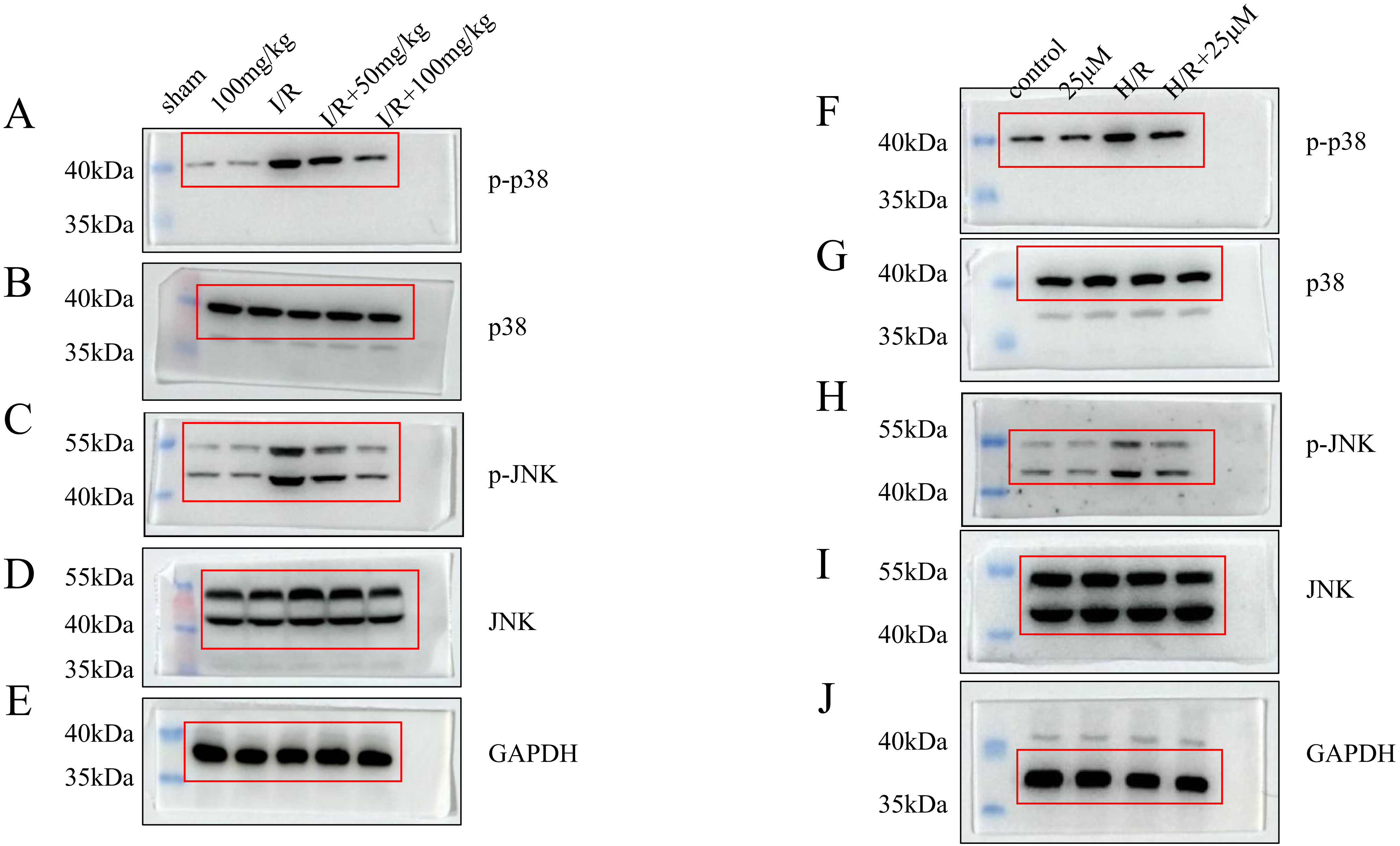

(A) p-p38 protein bands in Figure 5A; (B) p38 protein bands in Figure 5A; (C) p-JNK protein bands in Figure 5A; (D) JNK protein bands in Figure 5A; (E) GAPDH protein bands in Figure 5A; (F) p-p38 protein bands in Figure 5D; (G) p38 protein bands in Figure 5D; (H) p-JNK protein bands in Figure 5D; (I) JNK protein bands in Figure 5D;(J) GAPDH protein bands in Figure 5D.

Figure S5

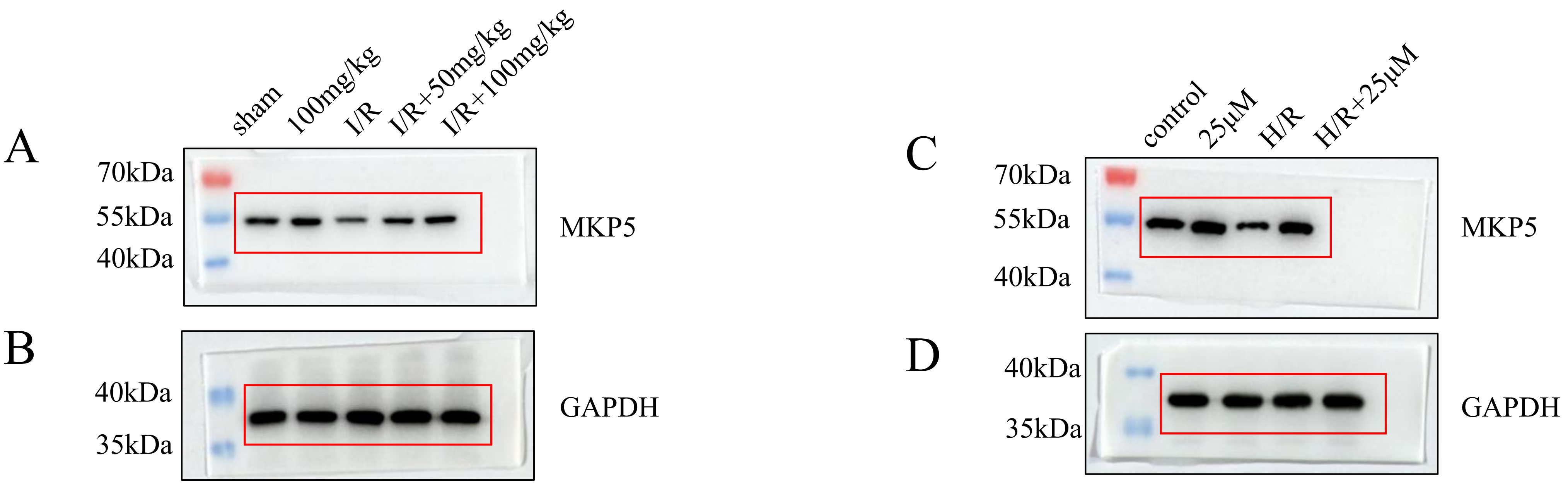

(A) MKP5 protein bands in Figure 6C; (B) GAPDH protein bands in Figure 6C; (C) MKP5 protein bands in Figure 6E; (D) GAPDH protein bands in Figure 6E.

Figure S6

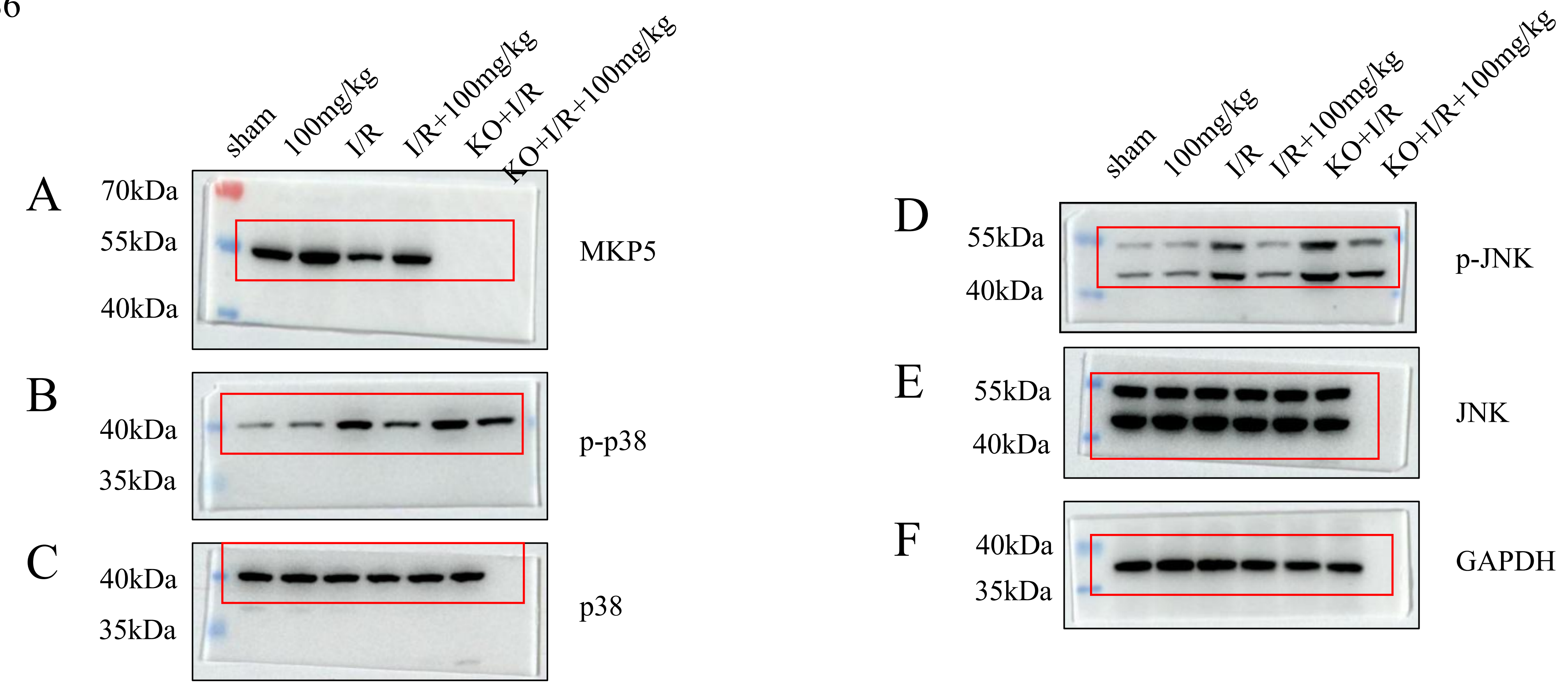

(A) MKP5 protein bands in Figure 7K; (B) p-p38 protein bands in Figure 7K; (C) p38 protein bands in Figure 7K; (D) p-JNK protein bands in Figure 7K; (E) JNK protein bands in Figure 7K; (F) GAPDH protein bands in Figure 7K.
