# Supplementary material for: 6-Gingerol attenuates hepatic ischemia/reperfusion injury through regulating MKP5-mediated P38/JNK pathway
Source: Sci Rep. 2024 Apr 2;14:7747. doi: 10.1038/s41598-024-58392-1 (PMC10987508; doi:10.1038/s41598-024-58392-1)
Supplement: Supplementary file 2 — Supplementary Information 2. [file 41598_2024_58392_MOESM2_ESM.docx]

**Generation of MKP5*^-/-^* mice**

A MKP5^-/-^ mouse model based on a C57BL/6 background was generated by CRISPR/Cas9 mediated gene-specific knockout as previously described ^[1, 2]^ ,and the mouse genotype was identified by DNA sequencing and PCR. The transcript targeted by the MKP5 knockout strategy is Ensembl number: ENSMUSG00000039384. The selected knockdown region is exon 2 region, and sgRNA target sites are selected in the upper and downstream sequences of the EXON 2 region. The sgRNA target sequence information is as follows: sgRNA 1: 5 '-TATGCAGAGGTCTTCCCCCGGGG-3', sgRNA 2: 5 '-AAATAATGTCCTGATCAGCTAGG-3'. sgRNA and Cas9 mRNA were microinjected into C57BL /6J fertilized eggs, and the fertilized eggs in good condition after injection were selected and transplanted into the oviducts of pseudopectic female mice to give birth to F0 generation mice naturally. The tails of 2-week-old first-born mice produced by prokaryotic injection were clipped, and genomic DNA was extracted for PCR amplification, with upstream primer: 5'-GGCTGAACTTGGATGCATGTCTC-3' and downstream primer: 5'-ACAGAGCTACCAGAGAAGGCATG-3'. The PCR amplification system and amplification procedure were as follows:

Table1 PCR amplification system for MKP5 genomic DNA

| PCR Reaction |  |
| --- | --- |
| 2×Taq Master Mix | 7.5 µL |
| upstream primer (10 µM) | 0.3 µL |
| downstream primer (10 µM) | 0.3 µL |
| Genomic DNA (20 ng/µL) | 1.0 µL |
| Add H2O up to | 15 µL |

Table 2 Amplification procedure for MKP5 genomic DNA

| Stage 1 | Rep: 1 | 94℃ | 5 min |
| --- | --- | --- | --- |
| Stage 2 | Reps: 35 | 94℃ | 30 sec |
|  |  | 55℃ | 30 sec |
|  |  | 72℃ | 35 sec |
| Stage 3 |  | 72℃ | 10 min |

The following figure shows the results of mouse DNA identification


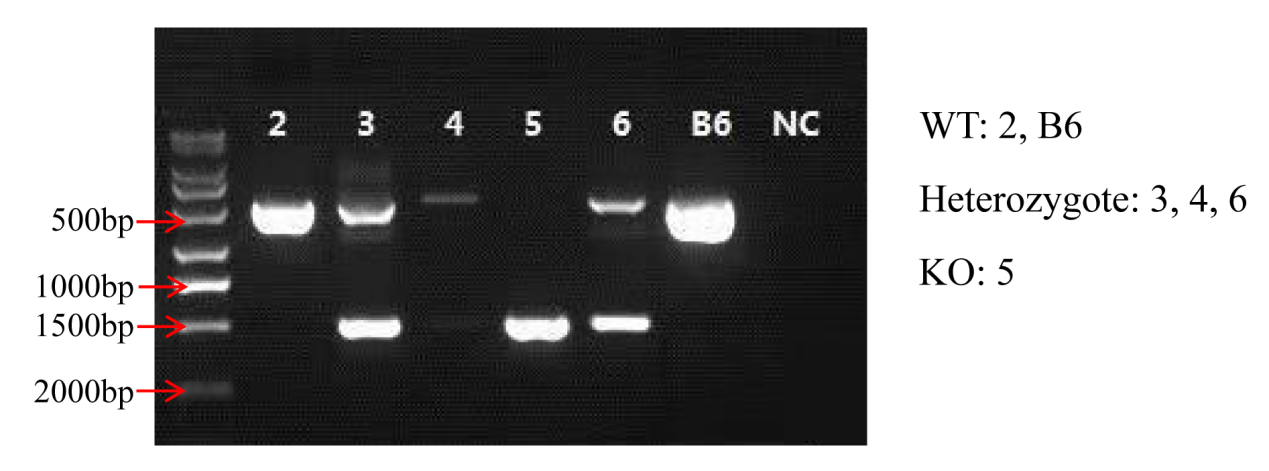


**Molecular docking between** **6-Gingerol and MKP5**

The 2D structure of 6-Gingerol was drawn using chemdraw 2D (version 19.0) and subsequently imported into Chemdraw 3D (version 19.0) and energy minimisation was carried out using MM2 module to get the advantageous conception of lowest energy and saved as mol2 file. The MKP5 protein structure (AF-Q9ESS0) was downloaded from AlphaFold Protein Structure Database and subsequently visualised using pymol respectively and then saved as pdbqt for ligand and receptor respectively using Mgtools (version 1.5.6) by de-watering, hydrogenation, calculating charge, merging non-polar hydrogens, etc.The ligand and receptor were docked using Autodock vina (version 1.1.2), the higher scoring conception was taken and visualised using PyMOL (version 2.5.2) and discovery studio (version 3.0).

The docking calculation result show that the binding affinity of 6-gingerol and MKP5 is -5.6 kcal/mol, indicating that Gingerol can spontaneously bind with MKP5 protein. 6-gingerol mainly interacts with MKP5 proteins through hydrogen bonding, hydrophobicity, and π-Stacking (perpendicular). Functional groups in 6-gingerol can form hydrogen bond interactions with amino acids PHE181, HIS194, ASN196 and ARG220 in MKP5 protein, while functional groups in Gingerol can form hydrophobic interactions with amino acids ASN196 and LEU457 in MKP5 protein. The benzene functional groups in 6-Gingerol can interact with PHE181 amino acids in MKP5 protein in π-Stacking (perpendicular), which are the main forces that promote compound binding to the active site.


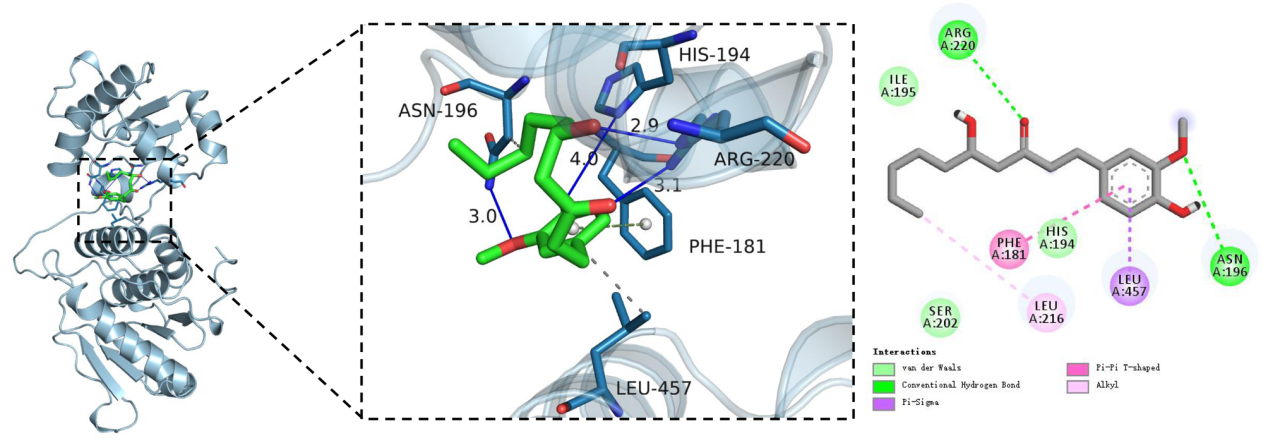


Schematic diagram of molecular docking of 6-Gingerol and MKP5.

**References**

[1] Chao T, Liu Z, Zhang Y, et al. Precise and Rapid Validation of Candidate Gene by Allele Specific Knockout With CRISPR/Cas9 in Wild Mice[J]. Front Genet, 2019,10:124.

[2] Huang R, Guo G, Lu L, et al. The three members of the Vav family proteins form complexes that concur to foam cell formation and atherosclerosis[J]. J Lipid Res, 2019,60(12):2006-2019.
